# Comparative analysis of doublet chemotherapy regimens plus bevacizumab in patients with recurrent ovarian cancer

**DOI:** 10.1097/MD.0000000000036750

**Published:** 2024-01-05

**Authors:** Ni̇jat Khanmammadov, İzzet Doğan, Necla Simay Okay, Abdulmunir Azizy, Pinar Saip, Adnan Aydiner

**Affiliations:** a Department of Medical Oncology, Istanbul University Institute of Oncology, Istanbul, Turkey; b Department of Internal Medicine, Istanbul University Faculty of Medicine, Istanbul, Turkey.

**Keywords:** bevacizumab, chemotherapy, metastasis, ovarian cancer

## Abstract

Among all gynecological malignancies, ovarian cancer is the predominant cause of mortality. Hence, various chemotherapy protocols have been established for managing metastatic ovarian cancer cases. The present study aimed to assess and compare the efficacy of dual chemotherapy regimens plus bevacizumab in patients diagnosed with recurrent platinum-sensitive epithelial ovarian cancer. This was a retrospective observational study. Data on the clinical, pathological, radiological, and treatment characteristics of the patients were recorded. Survival analyses were performed using the Kaplan–Meier method. Moreover, multivariate Cox regression analysis was conducted. Data of a total of 198 patients with a median follow-up duration of 18.7 months after bevacizumab treatment were analyzed. Serous carcinoma was found to be the most common pathological subtype in the analyzed patients, accounting for 85.8% of all cases. In total, 46.5% (n = 92), 38.4% (n = 76) and 15.2% (n = 30) patients had received gemcitabine plus carboplatin, paclitaxel plus carboplatin (PC), and gemcitabine plus cisplatin combined with bevacizumab, respectively. The complete response rate was 18.7%, partial response rate was 56.1%, stable disease rate was 6.6%, and progressive disease rate was 18.7%. The patients received bevacizumab treatment at a median of 9 cycles and doublet chemotherapy at a median of 7 cycles. The median progression-free survival was 11.9 (95% CI: 9.2–14.5) months, and the median overall survival (OS) was 24.7 (95% CI: 19.9–29.4) months. The results showed that a history of surgery prior to bevacizumab treatment was a significant factor affecting OS (*P* = .006). Patients who had received gemcitabine plus carboplatin with bevacizumab (28 months) had significantly better OS than patients who had received paclitaxel plus carboplatin (20.1 months) and gemcitabine plus cisplatin (17 months) (*P* = .009). Doublet chemotherapy regimens plus bevacizumab are safe and effective against recurrent platinum-sensitive epithelial ovarian cancer. Gemcitabine plus carboplatin with bevacizumab was superior to other treatment regimens in terms of OS outcomes.

## 1. Introduction

Ovarian cancer is the 8th most prevalent type of cancer among women and is the most lethal gynecological malignancy.^[1]^ Although ovarian cancer originates from a diverse array of tissues, approximately 95% of patients present with epithelial ovarian cancer (EOC).^[2]^

High-grade serous EOC, fallopian tube, and peritoneal carcinomas are unified clinical entities owing to their similar clinical characteristics and therapeutic approaches.^[3]^ Due to the absence of distinct symptoms specific to ovarian cancer, it is diagnosed at advanced stages in most cases.^[4]^ In recent years, considerable enhancements have been made in surgical techniques and chemotherapy, leading to better treatment modalities and reduced cancer-related mortality rates.^[5]^ Although initial therapy has high response rates, 80% of patients with advanced-stage EOC experience disease recurrence and death within 5 years.^[6]^ Management of recurrent ovarian cancer is based on the duration of progression from the last platinum-based treatment.^[7]^ Women with a platinum-free interval of <6 months (classified as platinum-resistant) or those with disease progression during platinum-based treatment (classified as platinum-refractory) have a poor prognosis, which is typically associated with a median overall survival (OS) of <12 months. Patients with disease recurrence exceeding 6 months from the last platinum treatment (referred to as platinum-sensitive disease) considerably benefit from subsequent platinum-based therapy.^[8,9]^ Several platinum-containing regimens, including combination treatments such as paclitaxel with carboplatin (PC), gemcitabine with carboplatin (GC), and pegylated liposomal doxorubicin with carboplatin, are effective for treating recurrent platin-sensitive EOC (PSI-EOC).^[10]^

Bevacizumab is a monoclonal antibody with humanized properties that specifically targets vascular endothelial growth factors. In previous phase 3 studies, the addition of bevcizumab to doublet platinum-based chemotherapy in recurrent PSI-EOC patients significantly improved both progression-free survival (PFS) and overall response rates (ORR). Following these results, the combination of doublet platinum-based chemotherapy and bevacizumab is used as the standard treatment regimen in these patients.^[11]^

In routine clinical practice, various factors, including treatment history, comorbidity, Eastern Cooperative Oncology Group performance status score, age, and potential treatment-related side effects, should be considered while selecting platinum-containing doublet chemoterapy regimens combined with bevacizumab for managing metastatic or recurrent PSI-EOC. Data comparing the efficacy of various doublet chemotherapy regimens are limited. Hence, the present study aimed to compare the efficacy of different doublet regimens with bevacizumab in patients with recurrent PSI-EOC.

## 2. Materials and methods

### 2.1. Patient information and data collection

The academic and ethical committees of our institution approved this retrospective cross-sectional study. This study was performed in accordance with the Declaration of Helsinki and the recommendations for good clinical practice. Patients who were monitored at an outpatient clinic of a specific oncology center from 2010 to 2022 were included. The participants were identified from the institution’s database. All patients diagnosed with recurrent PSI-EOC and treated with platin doublet chemotherapy regimens containing bevacizumab, as determined using the standard approach, were analyzed in this study. Patients who lacked adequate data for statistical analysis were excluded. Demographic and clinical data, including age at diagnosis, family history (International Federation of Gynecology and Obstetrics) stage, histology, adjuvant or neoadjuvant chemotherapy, number of bevacizumab cycles received, doublet chemotherapy regimens and number of cycles administered, radiotherapy treatments, surgeries, and toxicities, were collected from the medical database. These data were systematically recorded and documented for further analysis.

The treatment-related response was assessed clinically and radiologically at regular intervals of approximately 2 to 3 months. Based on the guidelines outlined in the Response Evaluation Criteria in Solid Tumors (RECIST 1.1), the treatment responses were categorized into the following 4 distinct subgroups: complete response (CR), partial response (PR), stable disease (SD), and progressive disease. These classifications were used to determine the best response achieved by patients in accordance with the specified criteria. ORR was calculated by combining the number of CR and PR. Meanwhile, the disease control rate was determined by including CR, PR, and SD cases. Treatment-related adverse events were recorded during each patient visit. The severity of these events was assessed and graded according to the Common Terminology Criteria for Adverse Events version 5. PFS was defined as the duration from the initiation of bevacizumab and doublet chemotherapy treatment to disease progression. OS was defined as the duration from the onset of recurrence to death for any reason. A univariate analysis was conducted to assess the impact of clinical and pathological parameters on OS. A multivariate analysis was performed using both the significant findings from the univariate analysis conducted in our study and the factors considered significant in the existing literature. Patient status was determined by cross-referencing with the death notification system of the Ministry of Health to ensure accurate and reliable information.

### 2.2. Statistical analysis

Statistical analyses were conducted using the Statistical Package for the Social Sciences software version 25. Continuous variables are presented as medians and the corresponding minimum and maximum values. Meanwhile, categorical variables are expressed as numbers and percentages. Survival curves were constructed using the Kaplan–Meier method. Univariate analysis was performed using the log-rank test. Multivariate analysis was conducted using the Cox regression model to assess the independent effects of various variables on the outcomes of interest.

## 3. Results

### 3.1. Characteristics of the patients and treatment modality

The present study included 198 patients diagnosed with primary ovarian cancer. The median age of the analyzed patients was 58 (28–88) years. In terms of the pathological characteristics of the patients, 85.8% (n = 170) presented with serous adenocarcinoma and 14.2% (n = 28) with other pathological subgroups (clear cell, endometrioid, and mucinous). Table 1 shows the clinical and pathological features of the patients. Primary surgery was performed in 96.5% (n = 191) of patients before bevacizumab treatment. In terms of perioperative chemoterapy, 4% (n = 8) received neoadjuvant treatment, 55.1% (n = 109) received adjuvant treatment, and 37.4% (n = 74) received neoadjuvant plus adjuvant treatment. While 91% (n = 182) of the patients were treated with paclitaxel and carboplatin as adjuvant and neoadjuvant therapy, the remaining few were treated with gemcitabine instead of paclitaxel due to comorbidities (e.g., risk of neuropathy). Seven patients, who accounted for 3.5% of all patients, did not receive perioperative chemotherapy. Approximately 40.4% (n = 80) of patients received palliative chemotherapy prior to bevacizumab administration. Regarding the chemotherapy regimens used in conjunction with bevacizumab in the study population, 38.4% (n = 76), 46.5% (n = 92), and 15.2% (n = 30) received PC, GC, and gemcitabine with cisplatin (GP) regimens, respectively. The median number of doublet chemotherapy cycles used in combination with bevacizumab was 7 (1–50). The average number of bevacizumab cycles was 9 (1–67).

**Table 1:** Clinical and pathological features of the patients.

Characteristics		n (%)
Age at diagnosis	<60 years ≥60 years	109 (55.1) 89 (44.9)
Family history for ovarian cancer	No	178 (89.9)
	Yes	20 (10.1)
Pathologic subtypes	Serous	170 (85.8)
	Others	28 (14.2)
Grade status	Grade 1–2	8 (4.0)
	Grade 3	189 (96.0)
BRCA mutation status	Not examined	145 (73.2)
	BRCA 1	21 (10.6)
	BRCA 2	2 (1.0)
	Negative	30 (15.2)
Stage at diagnosis	Stage 1-2	6 (3.0)
	Stage 3	155 (78.3)
	Stage 4	37 (18.7)
Sites of metastasis	Liver	87 (43.9)
	Peritoneal	188 (94.9)
	Lungs	37 (18.7)
	Others	24 (12)
The number of metastatic sites	≤2 sites	107 (54.0)
	>2 sites	91 (46.0)
Total number of surgeries prior to bevacizumab	No	7 (4.0)
	1	154 (77.3)
	2–4	37 (18.7)
Preoperative chemotherapy before bevacizumab	No	7 (3.5)
	Yes	191 (96.5)
Hormone therapy before bevacizumab	No	160 (80.8)
	Yes	38 (19.2)
Chemotherapy regimens combined with bevacizumab	Paclitaxel + carboplatin	76 (38.4)
	Gemcitabine + carboplatin	92 (46.5)
	Gemcitabine + cisplatin	30 (15.2)
After bevacizumab treatment	Chemotherapy	89 (44.9)
	Other (surgery, radiotherapy)	19 (9.6)

In terms of treatment responses, 18.7% (n = 37) and 56.1% (n = 111) of patients achieved CR and PR, respectively. Furthermore, 6.6% (n = 13) of patients had SD and 18.7% (n = 37) experienced progression (Table 2). Approximately 18.5% (n = 17) of patients in the GC plus bevacizumab (GCB) arm, 17% (n = 13) in the PC plus bevacizumab (PCB) arm, and 20% (n = 6) in the GP plus bevacizumab (GPB) arm presented with grade > 2 hypertension. In addition, grade ≥ 3 proteinuria was found in 6.5% (n = 6) of patients in the GCB group, 4% (n = 3) of patients in the PCB, and 6.6% (n = 2) of patients in the GPB group. The gastrointestinal system perforation rate of the GPB arm was not detected. However, it was 1% in the other arms. The fistula rates were 2.2% (n = 2) in the GCB group, 1.3% (n = 1) in the PCB group, and 3.3% (n = 1) in the GPB group (Table 3). Treatment with bevacizumab was discontinued in 78.3% (n = 155) of patients due to disease progression, 7.1% (n = 14) due to toxicity, and 9.6% (n = 19) due to insufficient treatment duration.

**Table 2:** Responses to bevacizumab plus doublet chemotherapy in recurrent PSI-EOC.

	Total n = 198 n (%)
*Response ratios* Complete response Partial response Stable disease Progressşon	3 (5) 26 (43) 11 (18) 20 (33)
Objective response ratio Disease control ratio	29 (48) 40(67)

**Table 3:** Grade > 2 side effects of bevacizumab + dual chemotherapy.

Variables	GCB n = 92 n (%)	PCB n = 76 n (%)	GPB n = 30 n (%)
Hypertension	17 (18.5)	13 (17)	6 (20)
Proteinuria	6 (6.5)	3 (4)	2 (6.6)
GIS perforation	1 (1.1)	1 (1.3)	0
Fistula	2 (2.2)	1 (1.3)	1 (3.3)
Thromboembolic events/hemorrhage	5 (5.5)	4 (5.3)	2 (6.8)
Febrile neutropenia	3 (3.3)	3 (4)	1 (3.3)
RPLS	0	0	0
Congestive heart failure	1 (1.1)	0	1 (3.3)

### 3.2. Survival outcomes

**Figure 1.**
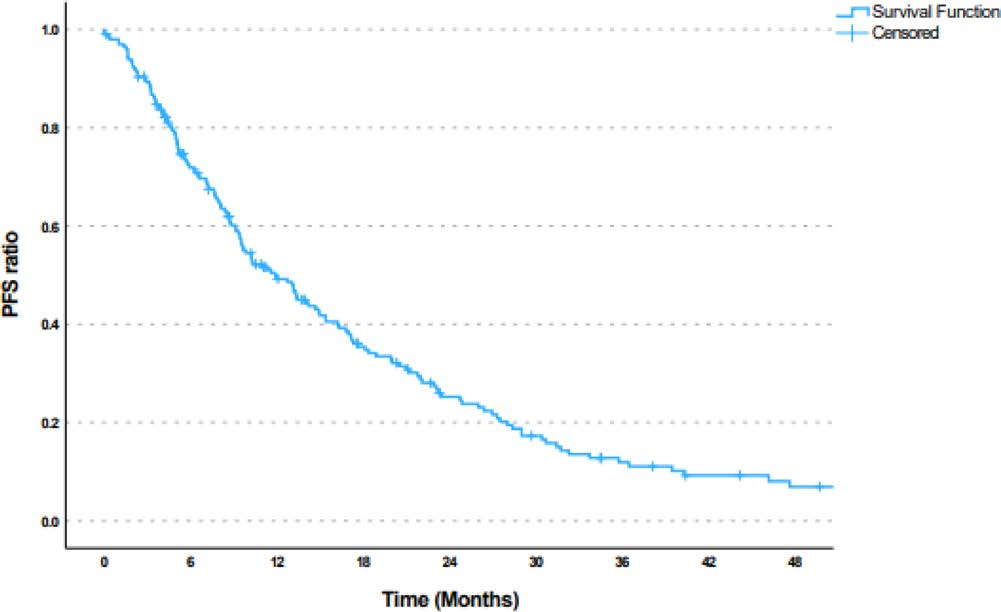
Kaplan–Meier curve of progression-free survival in patients with metastatic ovarian cancer treated with doublet chemotherapy plus bevacizumab.

**Figure 2.**
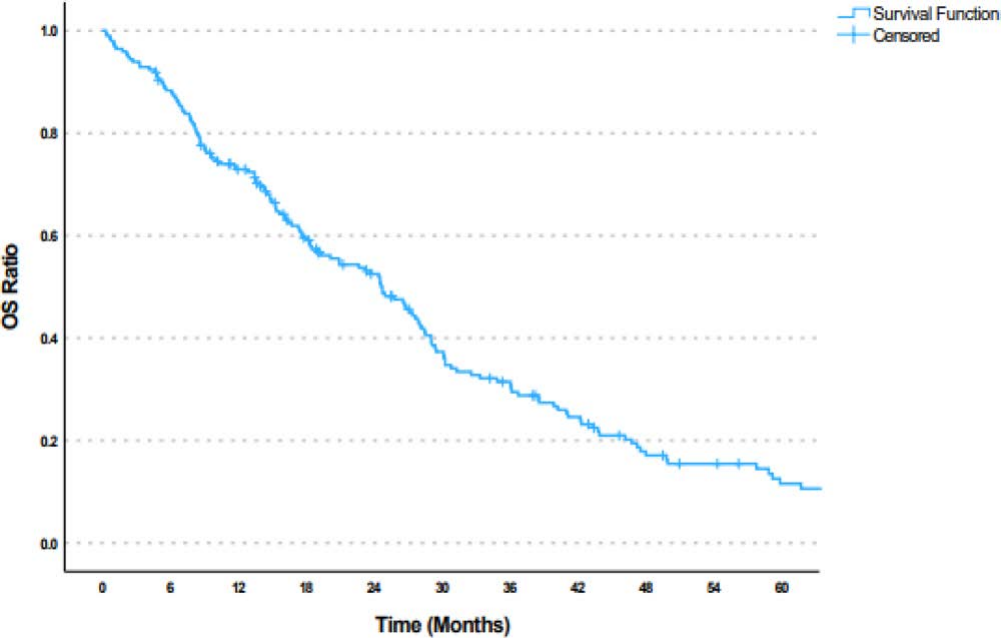
Kaplan–Meier curve of overall survival in patients with metastatic ovarian cancer treated with doublet chemotherapy plus bevacizumab.

**Figure 3.**
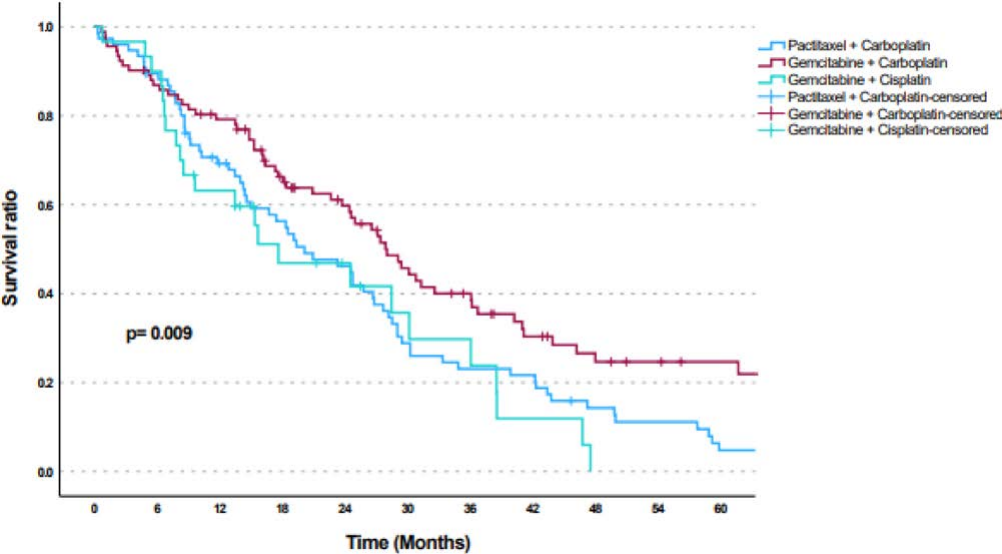
Kaplan–Meier curve of overall survival based on chemotherapy regimens with bevacizumab.

The median follow-up period after bevacizumab treatment was 18.7 months. The median follow-up period for PFS was 9.6 months. The median PFS was 11.9 (95% CI: 9.2–14.5) months (Fig. 1), and the median OS was 24.7 (95% CI: 19.9–29.4) months (Fig. 2). Moreover, there was no statistically significant difference in terms of survival rates according to age, pathology type, number of metastatic regions, grade, initial stage, perioperative chemotherapy, pre-bevacizumab radiotherapy, and hormone therapy status (*P* = .981, > 0.05). The survival rates of patients who underwent primary surgery before bevacizumab were substantially higher than those who did not (*P* = .001, < 0.01). Compared with regimens used with bevacizumab, the survival rate of patients receiving GC (28 months) was significantly higher than that of patients receiving PC (20.1 months) and GP (17 months) (*P* = .009, < 0.01) (Fig. 3). The hazard ratio (HR) for mortality in patients receiving GC was 0.633 (95% CI: 0.436–0.920), indicating a lower risk of mortality compared with other regimens (Tables 4 and 5).

**Table 4:** Univariate analysis for survival analysis.

		Total N	Ex N	Survivors N	Survival rate (60 month)	*P-value*
Age at diagnosis	<60	36	29	7	19%	.981
	≥60	24	23	1	4%	
Pathologic subtype	Seroz	170	128	42	24.7%	.630
	Others	28	25	3	10.7%	
Grade status	Grade 1	4	2	2	50.0%	.488
	Grade 2	4	4	0	0.0%	
	Grade 3	189	146	43	22.8%	
Stage at diagnosis	Stages 1–2	6	4	2	33.3%	.260
	Stage 3	155	118	37	23.9%	
	Stage 4	37	31	6	16.2%	
Primary surgery before bevacizumab treatment	No	7	7	0	0.0%	**.001**
	Yes	191	146	45	23.6%	
Liver metastasis	No	111	86	20	22.5%	.390
	Yes	87	67	20	23.0%	
Peritoneum metastasis	No	10	9	1	10.05	.121
	Yes	188	144	44	23.4%	
Lung metastasis	No	161	124	37	23.0%	.233
	Yes	37	29	4	21.6%	
Bone metastasis	No	189	145	44	23.3%	.159
	Yes	9	8	1	11.1%	
Number of metastatic sites	≤ 2 sites	107	84	23	21.5%	.172
	> 2 sites	91	69	22	24.2%	
	Yes	89	69	20	22.5%	
CT used in combination with bevacizumab	Paclitaxel + carboplatin	76	70	6	7.9%	**.009**
	Gemcitabine + carboplatin	92	60	32	34.8%	
	Gemcitabine + cisplatin	30	23	7	23.3%	
Denovo metastasis	No	161	122	39	24.2%	.074
	Yes	37	31	6	16.2%	
Perioperative CT	No	7	6	1	14.3%	**.029**
	Yes	191	147	44	23.0%	

Bold values indicates statistically significant.

**Table 5:** Multivariate cox regression analysis for overall survival.

		*P-value*	HR	95% CI	
				Lower	Upper
Age (<60 years vs ≥ 60)		.308	0.822	0.564	1.198
Pathologic subtype (serous vs non-serous)		.380	1.247	0.762	2.041
Grade	Grade 1		reference		
	Grade 2	.352	2.299	0.398	13.289
	Grade 3	.206	2.566	0.595	11.065
Denovo metastasis (yes vs no)		.158	1.422	0.872	2.320
Primary surgery before bevacizumab (yes vs no)		**.006**	**11.703**	**2.023**	**67.694**
Perioperatif CT (yes vs no)		.168	3.835	0.567	25.917
Liver metastasis (yes vs no)		.167	1.461	0.854	2.499
Lung metastasis (yes vs. no)		.945	0.981	0.563	1.708
CT in combination with bevacizumab					
Paclitaxel + carboplatin		**.024**	reference		
Gemcitabine + carboplatin		**.016**	**0.633**	**0.436**	**0.920**
Gemcitabine + cisplatin		**.632**	**1.125**	**0.690**	**1.834**

Multivariate analysis model *P*-value < .001. Bold values indicates statistically significant.

## 4. Discussion

The main findings of this study are as follows: In women with recurrent ovarian cancer who met the criteria for platinum-based treatment, the GCB regimen was associated with significantly longer OS compared with the PCB and GPB regimens (*P* = .009, < 0.01). In particular, the OS durations were 28 months in the GCB group, 20.1 months in the PCB group, and 17 months in the GPB group. To the best of our knowledge, this is the first trial comparing 3 different bevacizumab containing chemotherapy regimens against recurrent PSI-EOC.

Platinum-based doublet chemotherapy with bevacizumab has been reported to improve RR, PFS, and OS in patients with recurrent ovarian cancer. The phase III OCEANS trial aimed to assess the efficacy of bevacizumab in combination with GC versus GC plus placebo in patients with recurrent PSI-EOC. The bevacizumab containing regimen was associated with a higher ORR (79% vs 57%) and improved PFS (12.4 vs 8.4 months; HR: 0.48, 95% CI: 0.39–0.61). However, no significant difference was noted in terms of median OS between the 2 treatment arms (33.6 vs 32.9 months; HR: 0.95; *P* = .65).^[12]^ The GOG 213 trial compared the use of PCB and PC plus placebo in patients with PSI-EOC. The results showed that the bevacizumab group had a significantly higher proportion of patients achieving an ORR (78%) than the chemotherapy alone group (59%). Addition of bevacizumab significantly prolonged PFS (14 vs 10.8 months) and OS (37 vs 43 months).^[13]^ In another phase 3 study by Pfistereret et al, pegylated liposomal doxorubicin (PLD) plus bevacizumab was compared with GCB. The median PFS of patients receiving a PLD-containing regimen was 13.3 months, and that of patients receiving a gemcitabine-based regimen was 11.6 months. In addition, the median OS of the PLD group was 31.9 months, and that of the standard gemcitabine group was 27.8 months.^[14]^ Similar to the OCEANS and GOG 213 trials, our research found that patients receiving bevacizumab plus doublet chemotherapy had a high ORR at 74.8%. The PFS in our study was consistent with that of randomized prospective studies, with a duration of 11.9 months. The median OS in our study was slightly lower than that of other studies. This difference may be attributed to the fact that our study was based on real-world data, whereas other studies were conducted on selected patient populations. This finding suggests that differences in patient characteristics and treatment patterns between real-world settings and controlled clinical trials influence the observed outcomes. Primary debulking surgery is fundamental in the treatment of ovarian cancer and is considered to influence OS.^[15]^ In our study, patients who did not undergo primary debulking surgery before receiving doublet chemotherapy plus bevacizumab had a significantly lower OS than those who did (*P* = .001, <.01).

In individuals diagnosed with PSI-EOC who have achieved PR or CR after platinum-based treatment, the poly(ADP-ribose) polymerase (PARP) inhibitor olaparib has been approved by the FDA for maintenance therapy, irrespective of the BRCA status. In addition to olaparib, other PARP inhibitors, such as niraparib and rucaparib, are associated with enhanced PFS compared with placebo in patients with recurrent PSI-EOC, regardless of germline BRCA 1/2 mutations.^[16]^

After updating the latest data from clinical studies, the FDA has requested that the maintenance use of niraparib and rucaparib in relapsed ovarian cancer be restricted to patients who have genomic BRCA mutations. This recommendation originated from the observed insufficient benefit in terms of OS in individuals who do not possess BRCA mutation.^[17,18]^

In the present study, patients who received doublet chemoterapy plus bevacizumab regimens generally showed good tolerance to the treatment. In total, 14 (7.1%) patients developed drug withdrawal due to treatment-related side effects. The rate of febrile neutropenia was similar in all 3 groups. Regarding bevacizumab-related side effects, the incidence rates of grade ≥ 3 hypertension and proteinuria were high in all 3 arms; this result was similar to that reported in a previous study.^[19]^ None of the patients in the GPB group had gastrointestinal system perforation, which is a serious side effect. However, 1.1% and 1.3% of patients in the PCB and GPB groups presented such complications. Similar to randomized phase 3 studies, the thromboembolic events/hemorrhage rates in the GPB, PCB, and GPB groups were 5.5%, 5.3%, and 6.8%, respectively.^[20]^

### 4.1. Study limitations

First, the absence of a PARP inhibitor in each treatment group in our trial and the missing information regarding BRCA mutation status is a limitation. Although BRCA testing and PARP inhibitors were not widely available a few years ago, current guidelines recommend BRCA testing for all patients diagnosed with EOC.

Second, the retrospective design of the study revealed heterogeneity in the patient group, resulting in missing out some data. Furthermore, since it was a single-center study, there was a potential risk of selection bias.

## 5. Conclusions

Considering the factors mentioned above, selecting a platinum-based doublet chemotherapy regimen that can be used in conjunction with bevacizumab therapy for recurrent PSI-EOC is a concern. The present study showed that the GCB regimen with similar side effects was associated with higher OS compared with the PCB and GPB regimens. Therefore, our results can make a valuable contribution to the existing literature.

## Author contributions

**Conceptualization:** Nijat Khanmammadov, Izzet Dogan, Necla Simay Okay, Abdulmunir Azizy, Pinar Saip, Adnan Aydiner.

**Data curation:** Nijat Khanmammadov, Izzet Dogan, Necla Simay Okay, Abdulmunir Azizy.

**Formal analysis:** Nijat Khanmammadov, Izzet Dogan, Necla Simay Okay, Abdulmunir Azizy.

**Funding acquisition:** Nijat Khanmammadov.

**Investigation:** Nijat Khanmammadov.

**Methodology:** Nijat Khanmammadov, Izzet Dogan, Necla Simay Okay, Abdulmunir Azizy, Pinar Saip, Adnan Aydiner.

**Project administration:** Nijat Khanmammadov, Izzet Dogan, Pinar Saip, Adnan Aydiner.

**Resources:** Nijat Khanmammadov, Necla Simay Okay, Abdulmunir Azizy.

**Software:** Nijat Khanmammadov, Necla Simay Okay, Abdulmunir Azizy.

**Supervision:** Nijat Khanmammadov, Izzet Dogan, Pinar Saip, Adnan Aydiner.

**Validation:** Nijat Khanmammadov, Pinar Saip, Adnan Aydiner.

**Visualization:** Nijat Khanmammadov.

**Writing – original draft:** Nijat Khanmammadov, Izzet Dogan, Necla Simay Okay, Abdulmunir Azizy.

**Writing – review & editing:** Nijat Khanmammadov, Izzet Dogan, Pinar Saip, Adnan Aydiner.
